# New Bioconjugated Technetium and Rhenium Folates Synthesized by Transmetallation Reaction with Zinc Derivatives

**DOI:** 10.3390/molecules26082373

**Published:** 2021-04-19

**Authors:** Jordi Borràs, Julie Foster, Roxana Kashani, Laura Meléndez-Alafort, Jane Sosabowski, Joan Suades, Ramon Barnadas-Rodríguez

**Affiliations:** 1 Departament de Química, Edifici C, Universitat Autònoma de Barcelona, 08193 Cerdanyola del Vallès, Catalonia, Spain; jborrasamoraga@gmail.com; 2 Barts Cancer Institute, Queen Mary University of London, John Vane Science Centre, Charterhouse Square, London EC1M 5P, UK; j.m.foster@qmul.ac.uk (J.F.); roxana.kashani@qmul.ac.uk (R.K.); j.k.sosabowski@qmul.ac.uk (J.S.); 3 Istituto Oncologico Veneto IRCCS, 35128 Padova, Italy; laura.melendezalafort@iov.veneto.it; 4 Unitat de Biofísica/Centre d’Estudis en Biofísica, Facultat de Medicina, Departament de Bioquímica i Biologia Molecular, Universitat Autònoma de Barcelona, 08193 Cerdanyola del Vallès, Catalonia, Spain

**Keywords:** technetium, carbonyl, folic, transmetallation, radiopharmaceutical, rhenium

## Abstract

The zinc dithiocarbamates functionalized with folic acid **2_Zn_** and **3_Zn_** were synthesized with a simple straightforward method, using an appropriated folic acid derivative and a functionalized zinc dithiocarbamate (**1_Zn_**). Zinc complexes **2_Zn_** and **3_Zn_** show very low solubilities in water, making them useful for preparing Tc-99m radiopharmaceuticals with a potentially high molar activity. Thus, the transmetallation reaction in water medium between the zinc complexes **2_Zn_** or **3_Zn_** and the cation *fac*-[^99m^Tc(H_2_O)_3_(CO)_3_]^+^, in the presence of the monodentate ligand TPPTS, leads to the formation of the 2 + 1 complexes *fac*-[^99m^Tc(CO)_3_(SS)(P)] bioconjugated to folic acid (**2_Tc_** and **3_Tc_**). In spite of the low solubility of **2_Zn_** and **3_Zn_** in water, the reaction yield is higher than 95%, and the excess zinc reagent is easily removed by centrifugation. The Tc-99m complexes were characterized by comparing their HPLC with those of the homologous rhenium complexes (**2_Re_** and **3_Re_**) previously synthesized and characterized by standard methods. Preliminary in vivo studies with **2_Tc_** and **3_Tc_** indicate low specific binding to folate receptors. In summary, Tc-99m folates **2_Tc_** and **3_Tc_** were prepared in high yields, using a one-pot transmetallation reaction with low soluble zinc dithiocarbamates (>1 ppm), at moderate temperature, without needing a subsequent purification step.

## 1. Introduction

The bioconjugation of biomolecules to metallic fragments is an important topic with a wide range of potential applications, in particular, the development of new metal drugs for diagnosis and therapy [1,2,3]. In this context, we are developing a new approach based on a transmetallation reaction between zinc complexes and transition metal atoms [4,5,6]. The transmetallation reaction has been applied for many years as a useful reaction for preparing new compounds by the simple transfer of one ligand between different metals [7,8,9]. The Zn(II) complexes are particularly useful for this goal because it is well-known that the transmetallation reaction between Zn(II) and transition metals is usually favored from the thermodynamic point of view, due to the higher stability of bonds with a metal in d^n^ configuration (*n* = 1–9) compared with those to a metal in a d^10^ configuration as Zn(II) [4,5,6,10].

**Figure 1:**
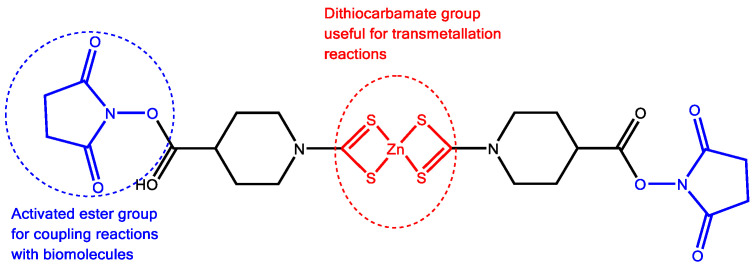
Molecular structure of **1_Zn_**.

Our strategy is based on using a functionalized Zn(II) dithiocarbamate complex (complex **1_Zn_**, Figure 1) which contains succinimidyl ester groups that make possible bioconjugation to a wide range of biomolecules [4,5,6]. This complex can be considered a useful tool for the bioconjugation of transition metals because it can be conjugated to biomolecules with a very simple experimental procedure. The obtained zinc complex allows for the formation of the bioconjugated complex with the desired transition metal by applying the simple transmetallation reaction. It should be highlighted that this compound has already been employed by another research group for preparing bioconjugated gold complexes [11], which is a very different goal to our research objectives.

Our approach is focused on preparing of Tc-99m complexes for radiopharmaceutical applications. The Tc-99m radionuclide is widely used for in vivo SPECT (single-photon emission computed tomography) imaging because it is easily accessible from ^99^Mo/^99m^Tc generators, it shows an appropriate half-life (≈ 6 h) and it has very suitable emission properties and detectable gamma rays [12,13,14]. A key point that should be kept in mind in the radiopharmaceutical chemistry of bioconjugated complexes with Tc-99m is that the concentration of this radioactive metal in the labeling reactions is very low (10^−6^–10^−8^ M), and consequently, although the concentration of the derivatized biomolecule used in the labeling reaction to form the corresponding metal complex could be very low, it is usually several orders of magnitude higher than the concentration of the radioactive metal [15]. Hence, to avoid receptor site saturation, the concentration of the unlabeled derivatized biomolecule should be as low as possible at the end of the process. Using Zn-dithiocarbamates is useful for this goal because they show very low solubilities in water, so the concentration of unlabeled biomolecule stays very low in the reaction medium (water) and the insoluble Zn-dithiocarbamate can be easily removed by filtration. In addition, Zn-dithiocarbamates are useful compounds for transmetallation reactions, a property that has been reported for different applications [4,5,6,10,11,16].

**Figure 2:**
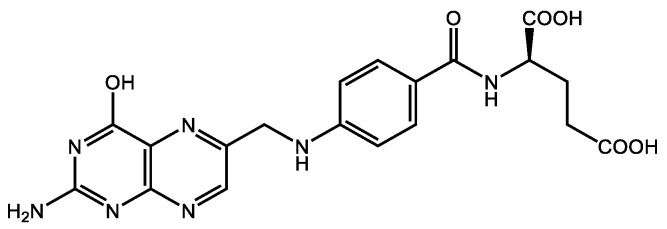
Folic acid.

In previous papers, we have shown the viability of this approach with the homologous rhenium complexes and with Tc-99m in the usual experimental conditions employed for the preparation of radiopharmaceuticals [4,5,6]. In this paper we have studied the viability of this approach for preparing the folate derivatives of rhenium and technetium-99m for radiopharmaceutical applications. Folic acid (Figure 2) is a vitamin (B9) necessary for cell growth because it is involved in the synthesis of purine and thymine nucleotides and the synthesis of the amino acid methionine [17,18]. The fast growth and proliferation of cancer cells requires a high consumption of folic acid, and, for this reason, the folate receptors are over-expressed in many cancer cells (ovarian, breast, cervical, nasopharyngeal and colon) [19,20]. Consequently, different methods to label folic acid with Tc-99m have been studied by different research groups [21,22,23,24,25,26]. In the present paper, we report the preparation of two new Zn(II) complexes bioconjugated to folic acid, using two different linkers between the folic acid and the dithiocarbamate group. We studied the transmetallation reaction of these complexes with rhenium and technetium-99m. Preliminary biological studies of the new Tc-99m complexes were also made to analyze whether these compounds could lead to new radiopharmaceuticals with high molar activity.

## 2. Results

### 2.1. Zinc Complexes

**Figure 3:**
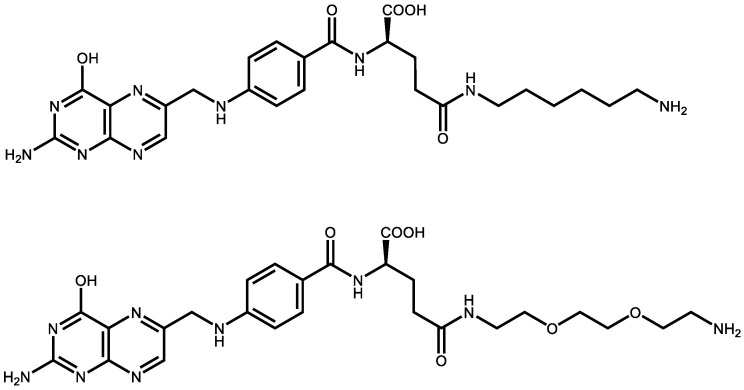
Folic acid derivatives.

The first step to obtain the bioconjugated zinc complexes was to derivatize the folic acid in order to obtain a molecule that contains a primary amino group, which can lead to the formation of the peptide bond with the activated ester groups of complex **1_Zn_**. Thus, the folic acid derivatives shown in Figure 3 were synthesized by using previously reported methods [27,28].

These organic compounds were characterized by the usual spectroscopic and spectrometric methods, and all data are consistent with those previously reported [27,28]. These compounds were chosen because they contain both an amine group that allows the biomolecule to be linked with complex **1_Zn_**, and a spacer that is useful to distance the folate group from the metal center, to limit the possible steric hindrance of the metallic center in the interaction with the folate receptor. Furthermore, we chose two derivatives with different polarities (see Figure 3). The top molecule contains an alkylic chain, and the bottom molecule contains a polyether chain. The use of two different linkers is interesting to compare the behavior of the possible radiopharmaceuticals in vivo, since it is well-known that more polar compounds show a lower interaction with blood proteins and favor elimination by renal excretion [24,29,30].

**Scheme 1:**
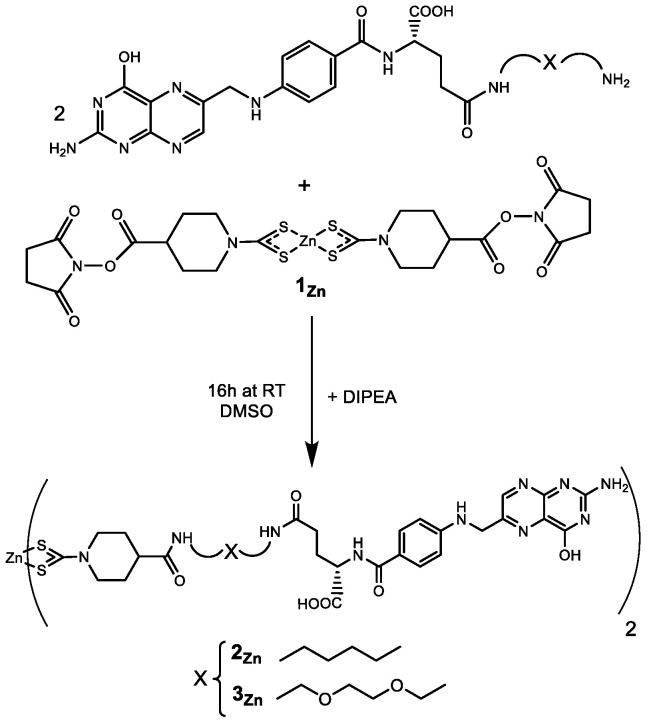
Synthesis of zinc complexes bioconjugated with folic acid (**2_Zn_** and **3_Zn_**).

The next step was the reaction of the two compounds of Figure 3 with the zinc complex **1_Zn_** to yield the new zinc complexes bioconjugated with folic acid **2_Zn_** and **3_Zn_**, as shown in Scheme 1.

These reactions were performed in similar experimental conditions to those previously reported [4,5]. The only modifications were that the solvent was changed to DMSO (dimethyl sulfoxide) because folic acid shows low solubility in DMF and the reaction time was longer. The final products **2_Zn_** and **3_Zn_** were easily separated from unreacted folic acid because the new zinc complexes are less soluble in water than folic acid. The two new complexes were characterized by the usual spectroscopic and spectrometric methods (^1^H, ^13^C NMR, IR and HRMS can be found as Supplementary Materials). Thus, the ^1^H NMR spectra are consistent with the formation of the bioconjugated zinc complexes, showing the characteristic signals of the isonipecotic ring after bioconjugation (4.83–4.86 ppm, assigned to the two equatorial protons) and the absence of the signals of the reagent **1_Zn_**. Furthermore, the presence of the characteristic aromatic signals of folic acid fragment and the absences of the signal of the primary amine group are in accordance with the hypothesis that the folic derivative has reacted and it is not present as a contaminant in the final products. The ^13^C NMR spectra are also consistent with the formation of the two new complexes, showing the characteristic signal of the dithiocarbamate group at 203.2 ppm and all expected signals of the different organic groups. Finally, the elemental analysis is in accordance with the calculated stoichiometry, and the analysis of the two compounds by ESI-HRMS shows the expected signals for **2_Zn_** (*m*/*e*; 787.1798; [M] − L^−^ − 2H^+^) and **3_Zn_** (*m*/*e*; 1579.4329; [M] − H^+^) in agreement with the theoretical calculated values (787.1792 and 1579.4293, respectively).

### 2.2. Rhenium Complexes

Before studying the transmetallation reaction with Tc-99m, it is necessary to synthesize and characterize the homologous rhenium complexes. Tc-99m complexes are prepared at a high dilution of Tc-99m: The concentration is so low (10^−6^–10^−8^ M) that it is not possible to use standard methods to analyze the complexes. However, it is well-known that they can be characterized by comparing their retention times in HPLC with those of their homologues rhenium complexes because they show similar retention times [12,13,14,31]. The *fac*-[Re(CO)_3_Br_3_]^2−^ reagent was chosen for this purpose because it is useful for synthesizing rhenium carbonyl complexes homologues of the Tc-99m complexes obtained with the cation *fac*-[^99m^Tc(H_2_O)_3_(CO)_3_]^+^ [32,33]. This Tc-99m complex is particularly useful for preparing new radiopharmaceuticals because the easy substitution of the labile water molecules leads to very inert and stable Tc-99m complexes that are useful for radiopharmaceutical purposes [32,33].

**Scheme 2:**
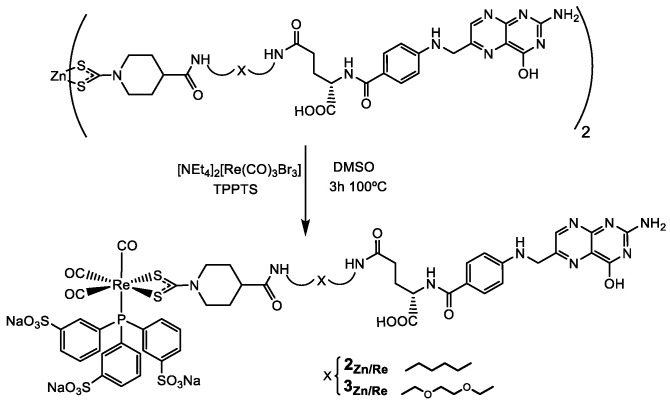
Synthesis of rhenium complexes **2_Re_** and **3_Re_**.

Complexes **2_Re_** and **3_Re_** were synthesized by using a similar method to that previously reported [4,5,6] for the transmetallation reaction between the bioconjugated zinc complexes, the rhenium metal complex [NEt_4_]_2_[Re(CO)_3_Br_3_] and the mono-dentated ligand TPPTS (triphenylphosphine trisulphonate), by means of the 2 + 1 reaction shown in Scheme 2. The co-ligand TPPTS was selected as monodentate ligand because it forms very stable complexes with Tc-99m and it increases the hydrophilicity of the complex, favoring its excretion by the renal-urinary system [34,35]. This monodentate ligand has also been used in other studies of Tc-99m with folate derivatives [22,23,25].

Complexes **2_Re_** and **3_Re_** were characterized by common spectroscopic and spectrometric methods (^1^H, ^13^C, ^31^P NMR, IR and HRMS can be found as Supplementary Materials). One of the most characteristic signals of the formation of the 2 + 1 complexes *fac*-[Re(CO)_3_(SS)(P)] is a signal in the ^31^P NMR spectra at around 17 ppm, assigned to the TPPTS ligand coordinated to the metal in this coordinative set, which is observed for both metal complexes. Another symptomatic signal, which shows that the transmetallation reaction has occurred, is the absence of the signal assigned to the equatorial protons of the isonipecotic group that are nearest to the dithiocarbamate fragment in the zinc complexes **2_Zn_** and **3_Zn_** (4.83–4.86 ppm), and the presence of a new signal with a shift of around 0.7 ppm (4.05–4.12 ppm). This shift has been previously observed after similar transmetallation reactions [4,5,6]. The IR spectroscopy is also very revealing because the signals of the rhenium carbonyl *fac*-[Re(CO)_3_Br_3_]^2−^ cannot be observed in the ν(CO) region, and they are substituted by the characteristic pattern of the *fac*-[Re(CO)_3_(SS)(P)] coordinative set [4,5,6,36]. Finally, ESI-HRMS shows the expected rhenium fragment in the negative region (*m*/*e*; **2_Re_**: 1541.1235 calculated for [M] − Na^+^, found 1541.1226; **3_Re_**: 775.0620 calculated for [M] − 2Na^+^, found 775.0614).

**Scheme 3:**
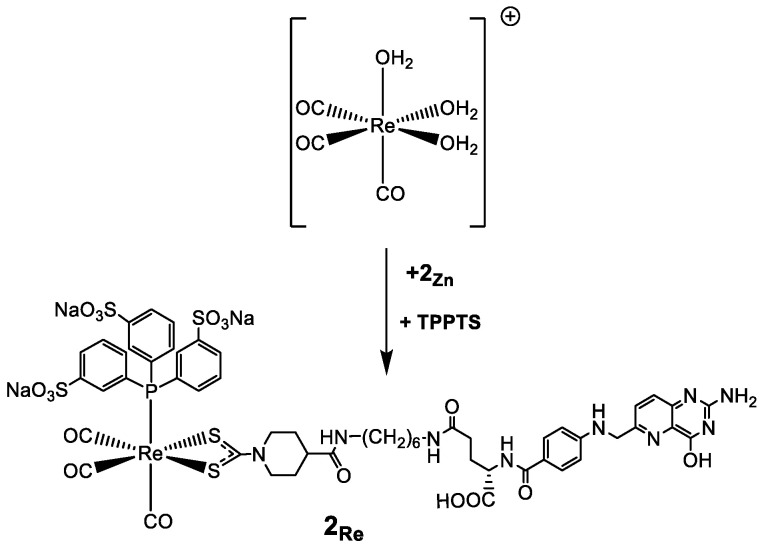
Synthesis of **2_Re_** in water medium at low concentration (20 ppm).

In order to obtain more information about the possibilities of the transmetallation reaction with rhenium, we made a study with experimental conditions close to those used for preparing radiopharmaceuticals. Thus, the transmetallation reaction was carried out in a water medium at a low concentration (20 ppm) of the cationic complex *fac*-[Re(H_2_O)_3_(CO)_3_]^+^ (Scheme 3). The analysis of the reaction mixture by ESI-HRMS, after separation of the unreacted **2_Zn_** complex by centrifugation, confirmed the formation of the expected bioconjugated rhenium complex **2_Re_**. These results indicate that this reaction is viable for preparing the homologous Tc-99m. They are also informative about possible studies for preparing the metal complexes with the β-emitting radionuclides Re-186/Re-188, which are useful for therapeutic purposes [29,37,38].

### 2.3. Technetium-99m Complexes

**Scheme 4:**
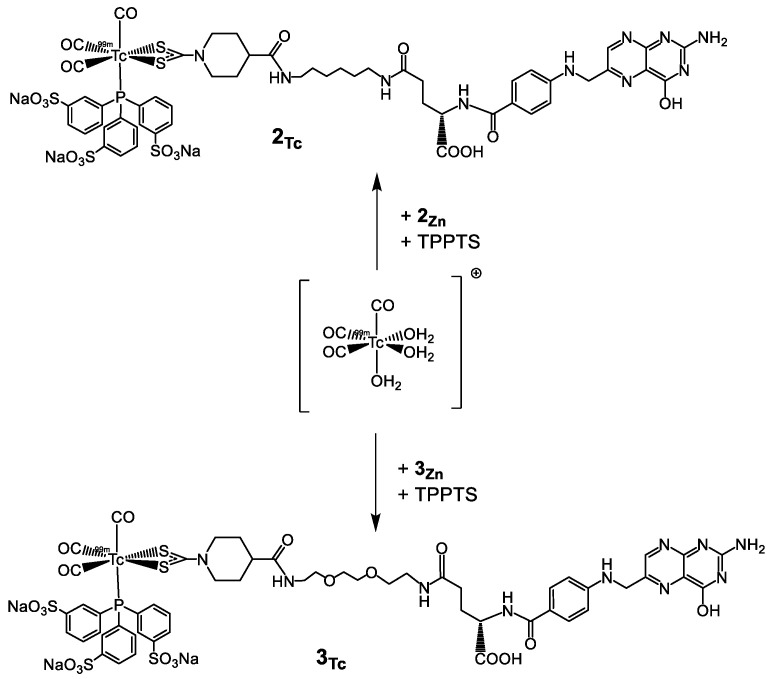
Synthesis of Tc-99m complexes **2_Tc_** and **3_Tc_**.

The Tc-99m complexes **2_Tc_** and **3_Tc_** (Scheme 4) were prepared under the experimental conditions commonly used for synthesizing Tc-99m radiopharmaceuticals. Reactions were carried out by adding a suspension of a small amount of the appropriate zinc complex (**2_Zn_** and **3_Zn_**, ~1 mg) and monodentate ligand (TPPTS) to an aqueous solution of [^99m^Tc(H_2_O)_3_(CO)_3_]^+^ and posterior heating at moderate temperatures (45–65 °C) for a short time (~25 min). After the suspension had cooled down, it was centrifuged, and the obtained water solution was ready to be used for the biological studies. The low reaction temperature and the short reaction time make this procedure appropriate for preparing Tc-99m compounds for radiopharmaceutical purposes, since long reaction times are incompatible with the decay of the radionuclides.

**Figure 4:**
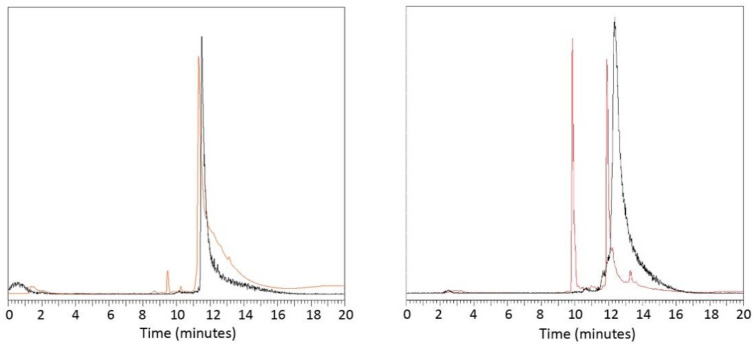
Chromatograms of Tc-99m complexes (black color, radiometric detector; (**left**) **2_Tc_**, (**right**) **3_Tc_**) and their homologous rhenium complexes (red color, UV detector; (**left**) **2_Re_**, (**right**) **3_Re_**). UV signal of **3_Re_** chromatograms was obtained from the complex prepared in DMSO at milligram level; therefore, only in this case is the peak at 10.0 min evident which corresponds to the presence of **3_Zn_**.

Figure 4 shows the chromatograms of **2_Tc_** and **3_Tc_** compared with those of the rhenium homologues **2_Re_** and **3_Re_**. As can be observed, the retention times of the homologous complexes are very similar, and the small differences are due to the different times that the sample needs for arriving to the two different detectors (UV-detector for rhenium complexes and gamma ray–detector for Tc-99m complexes). Hence, this result confirms that the homologous Tc-99m complexes **2_Tc_** and **3_Tc_** can be obtained by the simple transmetallation reaction with **2_Zn_** and **3_Zn_**. Furthermore, the yields of the radiosynthesis were higher than 95% in both cases, without any purification procedure. It was only necessary to centrifuge the reaction mixture to remove the excess of the insoluble zinc compound to obtain a water solution useful for biological studies. This is a simple procedure that could be used for preparing new radiopharmaceuticals [4,5,6].

In the Introduction, it was highlighted that a relevant point of the transmetallation reaction with zinc dithiocarbamate complexes is the low solubility of the zinc dithiocarbamates. This fact makes the bioconjugation of the Tc-99m possible in a medium with a very low concentration of the unlabeled biomolecule, increasing the molar activity (defined as the ratio between the radioactivity of a labeled biomolecule and the total amount of the biomolecule in moles [39]) of the potential radiopharmaceuticals. Thus, the water solubilities of complexes **2_Zn_** and **3_Zn_** were measured by inductively coupled plasma–optical emission spectrometry (ICP–OES), obtaining very low values for both complexes (**2_Zn_**, <0.2 ppm because is below the detection limit of the equipment; **3_Zn_**, 0.61 ppm). These results confirm that it is possible to prepare **2_Tc_** and **3_Tc_** with high yields in a medium with a very low concentration of the unlabeled biomolecule.

### 2.4. Serum Stability Studies

The stability of the coordinative set [^99m^Tc(CO)_3_(SS)(P)] in amino acid media and serum has been previously demonstrated [6]. However, the stability of **2_Tc_** and **3_Tc_** was assessed in serum to confirm their stability in physiological conditions before carrying out the in vitro studies.

**Figure 5:**
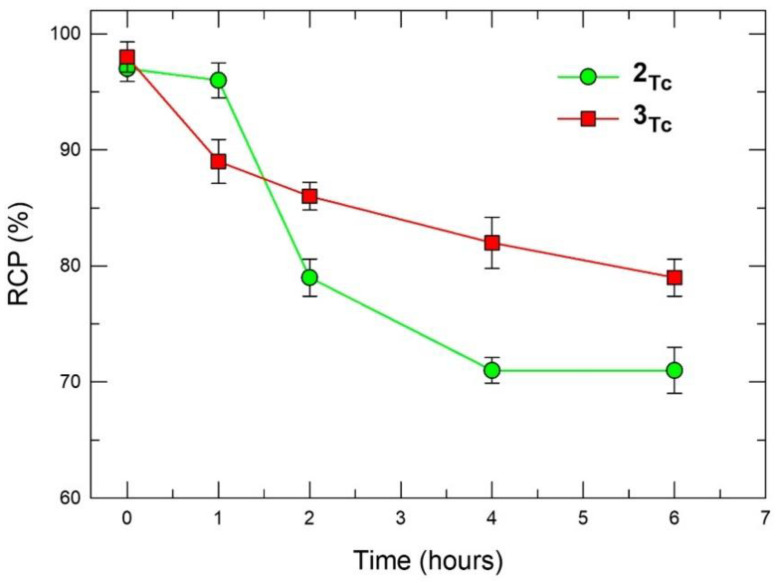
Stability study of complexes **2_Tc_** and **3_Tc_** in mouse serum (mean ± SD; *n* = 3).

Compounds **2_Tc_** and **3_Tc_** were added to fresh mouse serum and incubated at 37 °C for 6 h. Aliquots were removed at time points 1, 2, 3, 4 and 6 h, and the proteins were precipitated with acetonitrile. The RCP (radiochemical purity) of each aliquot was measured by RP-HPLC. The results (Figure 5) showed that **2_Tc_** and **3_Tc_** complexes have enough stability in serum to perform the in vitro and in vivo studies.

### 2.5. In Vitro Binding Assays

**Figure 6:**
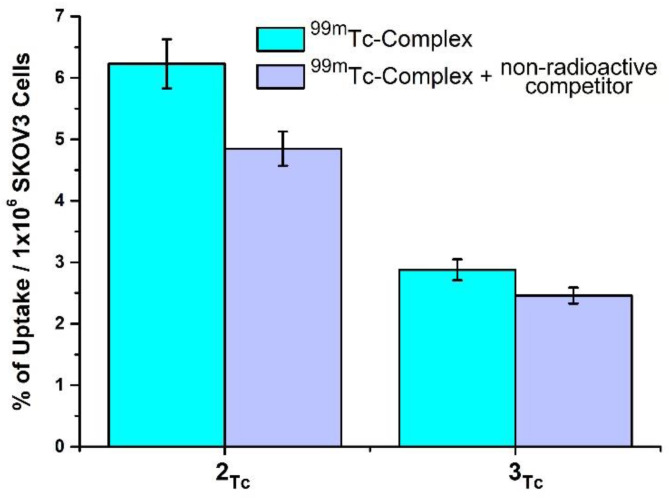
Results of binding assay for **2_Tc_** and **3_Tc_** carried out on SKOV3 ovarian cancer cells.

In vitro studies of the binding affinity of radiocompounds **2_Tc_** and **3_Tc_** with cells were carried out on SKOV3 ovarian cancer cells characterized by their overexpression of the folate receptor [40,41]. Cells were grown in media free of folic acid in order to avoid the blocking of the folate receptors. The comparison of blocked and unblocked binding assays carried out in parallel demonstrated only a minimal statistically significant difference (see Figure 6), which indicates low specific binding to the folate receptors. However, the non-specific binding determined on blocked cells was higher than that reported by other bioconjugated folate Tc-99m complexes [21,25].

### 2.6. Biodistribution Studies

Six-week-old female athymic mice bearing a SKOV3 tumor on the right flank (*n* = 3) were used for bio-distribution studies. Animals were injected with a high molar activity solution of **2_Tc_** or **3_Tc_** and sacrificed 4 h post-injection. Considering the activity injected and the solubilities of **2_Zn_** and **3_Zn_** the values of molar activity calculated were 6.7 × 10^2^ MBq/nmol and 2.1 × 10^3^ MBq/nmol for complexes **2_Tc_** and **3_Tc_**, respectively. These values are higher than 3.7 × 10^2^ MBq/nmol (10 Ci/μmol), the highest molar activity value reported for ^99m^Tc-radiopharmaceuticals as far as we know. [42].

The results in Table 1 show that both radiocomplexes were highly accumulated in the intestines due to the lipophilic nature of the ^99m^Tc(CO)_3_ core, which promotes the hepatobiliary excretion of the radiocompound [30,43,44]. Nevertheless, the incorporation of the polar ligand TPPTS to the **2_Tc_** and **3_Tc_** radiocompounds increases their hydrophilic character, which enhances their renal clearance. Consequently, the significant kidney uptake is due to both the presence of folate receptors in this organ [45] and the renal clearance of the complexes.

Tumor uptake is only from two to four times higher than muscle, which are quite low values to be considered good diagnosis agents. These results are consistent with the low specific binding to the folate receptors found in vitro and could be associated with the molecular structure of the complexes. In order to improve the specific binding and the tumor uptake, further studies involving changing the complex structure are necessary.

## 3. Materials and Methods

### 3.1. General

All reactions were performed under nitrogen, using standard Schlenk tube techniques. Complex **1_Zn_** [4], and folic acid derivatives [CAS 62682-21-7] [27] and [CAS 1099829-15-8] [28] were synthesized by using previously reported methods. The NMR spectra were recorded in the Servei de Ressonància Magnètica Nuclear at the Universitat Autònoma de Barcelona (UAB, Cerdanyola del Vallès, Spain), on a Bruker DPX-250, DPX-360 and AV400 instruments. Infrared spectroscopy, microanalyses and mass spectrometry measurements were performed by the Servei d’Anàlisi Química del Departament de Química (UAB, Cerdanyola del Vallès, Spain). Attenuated total reflection infrared spectra (ATR-IR) were registered in Tenson 72 equipment from Bruker. Microanalysis was performed with Thermo Fisher Scientific Flash EA 2000 CHNS equipment, and zinc concentrations were determined by inductively coupled plasma–optical emission spectroscopy (ICP–OES) with the equipment optima 4300DV System (Perkin-Elmer). High-resolution electrospray ionization mass spectrometry (ESI-HRMS) analyses were recorded by means of liquid chromatography equipment 1200 RR from Agilent Technologies, coupled with a microTOF-Q time-of-flight detector from the company Bruker Daltoniks.

### 3.2. Synthesis of ***2_Zn_***

Folic acid derivative [CAS 62682-21-7] (2.38 g, 4.4 mmol) was dissolved in DMSO (55 mL) by slightly heating. Complex **1_Zn_** (1.22 g, 1.8 mmol) and DIPEA (*N*,*N*-diisopropylethylamine; 1.7 mL, 9.9 mmol) were added to the previous solution, and the resulting suspension was stirred at room temperature, overnight. After the addition of 150 mL of THF, the product was precipitated as a yellow solid. It was collected by filtration and washed with a basic water solution (NaOH; pH 9–10) (4 × 15 mL) and acetone (2 × 20 mL). Yield: 2.37 g (85%). IR (ATR, cm^−1^): 3294 (N-H); 1604 (C=O); 3271 (N-H); 1490 (C-N, S_2_CN). ^1^H-NMR (DMSO-d_6_, δ in ppm): 8.64; 7.65; 6.66 (10H, ArH); 8.01; 7.81 (6H, RN*H*COR); 6.92 (s, 2H, ArN*H*-R); 4.86 (d, 4H, S_2_CNC*H*,eq); 4.49 (d, 2H, ArC*H_2_*NHAr); 4.31 (s, 2H, NHC*H*COOH); 3.02 (s, 4H, OCNHC*H_2_*CH_2_); 2.24–2.06 (m, 8H, COOHCHC*H_2_*C*H_2_*CO); 1.76 (s, 4H, S_2_CNCH_2_C*H*,eq); 1.59 (s, 4H, S_2_CNCH_2_C*H*,ax); 1.36–1.09 (16H, alkyl). ^13^C-NMR (DMSO-d6, δ in ppm): 203.2 (S_2_*C*N); 175.5 (*C*OOH); 173.8 (R*C*ONH, isonipecotic); 172.3 (CH_2_CH_2_*C*ONH); 166.6–166.1 (HN*C*OCN, pteridine ring) and *C*ONH (aminobenzoic acid); 156.6 (NH_2_*C*NHN, pteridine ring); 151.1 (HN*C*, aminobenzoic acid); 148.8 (NH*CC*HN, pteridine ring); 129.1 (HNCCH*C*HCCONH (aminobenzoic acid) and NHCO*C*N (pteridine ring)); 121.9 (Ar*C*CONH, aminobenzoic acid); 111.7 (HNC*C*HCHCCONH, aminobenzoic acid); 53.8 (NH*C*COOH); 51.0 (S_2_CN*C*H_2_); 46.3 (Ar*C*H_2_NHAr); 32.2–15.2 (alkyl). ESI-HRMS (negative mode, *m*/*z*): calcd. for C_32_H_39_N_10_O_6_S_2_Zn ([M] − L^−^ − 2H^+^), 787.1792, found 787.1798. Anal. Elem for C_64_H_82_N_20_O_12_S_4_Zn·3H_2_O: C, 48.9; H, 5.65; N, 17.5. Experimental: C, 48.8; H, 5.82; N, 17.5.

### 3.3. Synthesis of ***3_Zn_***

Folic acid derivative [CAS 1099829-15-8] (2.5 g, 4.4 mmol) was dissolved in DMSO (30 mL) by slightly heating. Complex **1_Zn_** (1.22 g, 1.8 mmol) and DIPEA (1.5 mL, 8.8 mmol) were added to the previous solution, and the resulting suspension was stirred at room temperature, overnight. After the addition of 95 mL of THF, the product was precipitated as a yellow solid. It was collected by filtration and washed with a basic water solution (NaOH; pH 9–10) (3 × 20 mL) and acetone (2 × 20 mL). Yield: 2.56 g (88%). IR (ATR, cm^−1^): 3282 (N-H); 1604 (C=O); 1510 (C-N, S_2_CN). ^1^H-NMR (DMSO-d_6_, δ in ppm): 8.64; 7.63; 6.65 (10H, ArH); 8.02; 7.93; (s, 6H, RN*H*COR)); 6.93; (s, 2H, ArN*H*R); 4.83 (d, 4H, S_2_CNC*H*,eq); 4.49 (d, 4H, ArC*H_2_*NHAr); 4.32 (s, 2H, NHC*H*COOH); 3.19 (b, 24H, OCNHC*H_2_*C*H_2_*OC*H_2_*C*H_2_*OC*H_2_*C*H_2_*NHCO); 2.30–2.17 (m, 8H, COOHCHC*H_2_*C*H_2_*CO); 1.79 (s, 4H, S_2_CNCH_2_C*H*,eq); 1.56 (s, 4H, S_2_CNCH_2_C*H*,ax). ^13^C-NMR (DMSO-d_6_, δ in ppm): 203.2 (S_2_*C*N); 174.0 (R*C*ONH, isonipecotic); 172.4 (CH_2_CH_2_*C*ONH); 166.6 (HN*C*OCN, pteridine ring; *C*ONH, amino-benzoic acid); 156.7 (NH_2_*C*NHN, pteridine ring); 151.4 (HN*C*, amino-benzoic acid); 149.0 (NH*CC*HN, pteridine ring); 129.5 (HNCCH*C*HCCONH, amino-benzoic acid; NHCO*C*N, pteridine ring); 121.8 (Ar*C*CONH, amino--benzoic acid); 111.9 (HNC*C*HCHCCONH, amino-benzoic acid); 69.9–69.5 (R*C*H_2_O*C*H_2_*C*H_2_O*C*H_2_R); 65.6 (OCNHCH_2_*C*H_2_O*C*H_2_*C*H_2_O*C*H_2_CH_2_NHCO); 53.8 (NH*C*COOH); 51.3 (S_2_CN*C*H_2_); 46.4 (Ar*C*H_2_NHAr); 29.0 (COOHCH*C*H_2_CH_2_CONH); 19.5 (S_2_CNCH_2_*C*H_2_); 15.8 (COOHCH*C*H_2_CH_2_CONH). ESI-HRMS (negative mode, *m*/*z*): calcd. for C_64_H_81_N_20_O_16_S_4_Zn ([M] − H^+^), 1579.4293, found 1579.4329. Anal. Elem for C_64_H_82_N_20_O_16_S_4_Zn·2H_2_O: C, 47.5; H, 5.36; N, 17.3. Experimental: C, 47.6; H, 5.54; N, 17.1.

### 3.4. Synthesis of ***2_Re_***

The precursor [NEt_4_]_2_[Re(CO)_3_Br_3_] (89 mg, 0.11 mmol) was dissolved in degassed DMSO (2.5 mL) and added to a solution of **2_Zn_** (100 mg, 66.1 μmol) in also degassed DMSO (2 mL). The red resulting mixture was heated at 110 °C for 1 h. Next, TPPTS (69 mg, 0.12 mmol) was added, and the resulting suspension was heated for two additional hours. After cooling down the reaction solution at room temperature, 75 mL of absolute ethanol:methanol (3:1) mixture was added to yield a yellow precipitate, which was then collected by centrifugation and washed with cold ethanol (3 × 10 mL). Yield: 127 mg (70%). IR (ATR, cm^−1^): 3328 (NH); 2017, 1919; 2017 (C≡O); 1604 (C=O); 1501 (C-N, S_2_CN). ^1^H NMR (DMSO-d_6_, δ in ppm): 8.65; 7.79–7.48; 6.65 (17H, ArH); 8.01; 7.81; 6.98; (s, 3H, RN*H*COR); 4.48 (d, 2H, ArC*H_2_*NHAr); 4.26 (s, 1H, NHC*H*COOH); 4.05 (dd, 2H, S_2_CNC*H*,eq); 3.02 (s, 4H, OCNHC*H_2_(*CH_2_*)*_4_C*H_2_*NHCO); 2.21 (s, 2H, S_2_CNCH_2_C*H*,eq); 1.82 (m, 4H, COOHCHC*H_2_*C*H_2_*CO); 1.59 (s, 2H, S_2_CNCH_2_C*H*,ax). ^31^P NMR (DMSO-d_6_, δ in ppm): 17.3, also observed 17.1. ^13^C NMR (DMSO-d_6_, δ in ppm): 193.1 (CO); 172.3 (CH_2_CH_2_*C*ONH); 166.9 (HN*C*OCN, pteridine ring; *C*ONH, amino-benzoic acid); 151.3 (HN*C*, amino-benzoic acid); 149.2–148.3 (NH*CC*HN, pteridine ring); 134.9–122.0 (TPPTS); 111.4 (HNC*C*HCHCCONH, amino-benzoic acid); 51.8 (S_2_CN*C*H_2_); 46.3 (Ar*C*H_2_NHAr); 29.4–25.5 (alkyl). ESI-HRMS (negative mode, *m*/*z*): calcd. for C_53_H_53_N_10_O_18_S_5_Na_2_PRe ([M] − Na^+^), 1541.1235, found 1541.1226. Retention time RP-HPLC (min): 11.4.

### 3.5. Synthesis of ***3_Re_***

The precursor [NEt_4_]_2_[Re(CO)_3_Br_3_] (127 mg, 0.16 mmol) was added to a solution of **3_Zn_** (150 mg, 0.095 mmol) in degassed DMSO (1 mL), and the resulting solution was heated at 110 °C for 1 h. At that point, TPPTS (98 mg, 0.17 mmol) was added to the previous red solution, which was then heated for an additional 2 h. At the end of the reaction, the solution was cooled at room temperature, and then absolute ethanol (10 mL) was added to the mixture to promote product precipitation, which was then collected by centrifugation and washed with cold ethanol (2 × 10 mL). Yield: 159 mg (70%). IR (ATR, cm^−1^): 3311 (NH); 2020, 1923; 1896 (C≡O); 1607 (C=O); 1510 (C-N, S_2_CN). ^1^H-NMR (DMSO-d_6_): 8.64; 7.82–7.45; 6.65 (13H, ArH); 7.98; 7.83; 6.98; (s, 3H, RN*H*COR); 4.48 (d, 2H, ArC*H_2_*NHAr); 4.37 (s, 1H, NHC*H*COOH); 4.12 (dd, 2H, S_2_CNC*H*,eq); 3.17 (s, 12H, OCNHC*H_2_*C*H_2_*OC*H_2_*C*H_2_*OC*H_2_*C*H_2_*NHCO); 2.23 (s, 2H, S_2_CNCH_2_C*H*,eq); 1.96–1.80 (m, 4H, COOHCHC*H_2_*C*H_2_*CO); 1.62 (s, 2H, S_2_CNCH_2_C*H*,ax). ^31^P-NMR (DMSO-d_6_, δ in ppm): 16.9, also observed 16.6. ^13^C-NMR (DMSO d_6_, δ in ppm): 149.1 (NH*CC*HN, pteridine ring); 129.5–121.9 (TPPTS); 112.4 (HNC*C*HCHCCONH, amino-benzoic acid); 69.3 (R*C*H_2_O*C*H_2_*C*H_2_O*C*H_2_R); 51.3 (S_2_CN*C*H_2_); 46.4 (Ar*C*H2NHAr); 27.9–25.6 (alkyl). ESI-HRMS (negative mode, *m*/*z*): calcd. for C_53_H_53_N_10_O_20_S_5_NaPRe ([M] − 2Na^+^), 775.0620, found 775.0614. Retention time RP-HPLC (min): 11.9.

### 3.6. Preparation of Tc-99m Complexes

Na [^99m^TcO_4_] was eluted and provided by CADISA and Barts Health NHS Trust from a ^99^Mo/^99m^Tc generator, using 0.9% saline solution. The precursor *fac*-[^99m^Tc(CO)_3_(H_2_O)_3_]^+^ was synthetized either with the Isolink kit (Coviden) or with an alternative method [46]. Briefly, in a 20 mL vial was added sodium potassium tartrate (15 mg), NaBH_4_ (6.5 mg) and Na_2_CO_3_ (6.0 mg), and then the vial was carefully sealed and flushed for 15 min with CO. Finally, 1 mL of NaTcO_4_ (37 MBq) was added and heated to 90 °C for 25 min. For both methods, after the heating period, the vial was cooled down to room temperature, and the pH was adjusted to approximately 7.0 with HCl 0.1 M. The radiochemical yield was evaluated with an RP-HPLC.

For the preparation of Tc-99m complexes **2_Tc_** and **3_Tc_**, 100 µL of *fac*-[^99m^Tc(CO)_3_(H_2_O)_3_]^+^ (10^−6^–10^−8^ M) was added to a suspension of the zinc dithiocarbamate compound (**2_Zn_** or **3_Zn_**) (150 µL, 10 mg/mL), previously sonicated for 15 min, with 10 µL of the solution of the TPPTS (10^−3^ M). The resulting suspension was heated to 40–65 °C for 25 min. Afterwards, the reaction mixture was cooled down, and the solid was removed by centrifugation. Retention time RP-HPLC (min): 11.5 (**2_Tc_**); 12.4 (**3_Tc_**). Radiochemical yields: 96% (**2_Tc_**); 98% (**3_Tc_**).

### 3.7. Reverse-Phase High-Performance Liquid Chromatography

The HPLC system featured at Barts Cancer Institute is a Beckman HPLC system Gold 128 with solvent module and a 166 UV detector module (monitoring at 280 nm). Radioactivity was detected by using a gamma-detector (Raytest). Compounds were separated on a Jupiter 5 µm C4 from company Phenomenex. Reverse phase analysis was carried out by using: (A) aqueous 0.1 M TEAP (triethylammonium phosphate solution) and (B) acetonitrile. Method: 0–5 min, 5% B; 3–15 min, 5–75% B; 15–20 min, 75% B; 20–25 min, 75–5% B; 25–28 min, 5% B. Flow rate: 1 mL min^−1^.

### 3.8. Determination of Solubilities of Zinc Compounds

A suspension in water (10 mL) of the appropriate zinc precursor (**2_Zn_** or **3_Zn_**) was heated at 40 °C for 1 h. Afterwards, the suspension was filtered through a 0.2 µm and centrifuged at 13,000 rpm for 20 min. Finally, the solution was diluted in 1% HNO_3_, and the zinc content was analyzed by means of inductively coupled plasma–optical emission spectrometry (ICP–OES).

### 3.9. Serum Stability Studies

Mice blood (4 mL) was centrifuged at 3000 rpm; the supernatant obtained (600 µL) was added to a 150 µL of the radiocompound solution (**2_Tc_** or **3_Tc_**) and incubated at 37 °C. At each time point (0, 1, 2, 4 and 6 h), aliquots of 150 µL were taken and added to 150 µL of acetonitrile, to precipitate the proteins. Each sample was stirred for 5 min and centrifuged. The supernatant was analyzed by RP-HPLC to evaluate the stability of the radiocompound.

### 3.10. Cell Culture

SKOV-3 cells (human ovarian adenocarcinoma) were cultured as monolayers at 37 °C in a humidified atmosphere containing 5% CO_2_. The study was performed with free-folate culture medium FFRPMI (modified RPMI, without folic acid, vitamin B12 and phenol red) supplemented with 10% (*v*/*v*) of FBS as the only source of folic acid. Cells were harvested in 75 cm^2^ flasks; when they achieve 80% of confluence, the medium was removed and cells were washed with the PBS buffer (10 mL). Then, 2 mL of trypsin-EDTA was added, and the flask was left in the incubator for 3 min, to remove cells from the flask wall. Finally, 4 mL of fresh media was added to deactivate the trypsin; cells suspensions were then diluted (1:3) and transferred into a new flask. This procedure was repeated approximately 2 or 3 times a week, depending on the dilution factor.

### 3.11. Binding Assay

To a PBS solution with 2.7 × 10^6^ cells in 0.5 mL, 250 µL (KBq) of the radiocomplex (**2_Tc_** or **3_Tc_**) was added, and then the mixture was incubated for 1.5 h at 37 °C. Then samples were centrifuged, and the pellets were washed three times with PBS. Blocking assay were carried out in parallel, adding to 2.7 × 10^6^ cells 250 µL radiocompound and 200 µL of folic acid (3 mM), at the same time, in order to block the folate receptors. Finally, the activity in all the pellets was measured with a gamma counter. The cell uptake of the radiocompound was given by the formula % uptake = (counts of cells/total counts) × 100. All the experiments were done in triplicate.

### 3.12. Biodistribution Studies

Three nude mice (6 weeks old) fed with a folate-free rodent diet were inoculated subcutaneously with a suspension of SKOV3 Cells (5 × 10^6^ cells in 100 μL PBS with 100 μL of Matrigel) into the right flank, 15–17 days before the study. Biodistribution studies were carried after injection of 200 µL of the radiocomplex (**2_Tc_** or **3_Tc_**) (7–12 MBq) into the tail vein. Animals were sacrificed 4 h post-injection; all the interested organs and tissues were explanted, weighted and measured with a gamma counter. The uptake was expressed as a percentage of the injected dose per gram of tissue. All experiments on animals were approved by the QMUL Animal Welfare and Ethical Review Body and were carried out under UK Home Office Project License PPL70/7493.

## 4. Conclusions

This study confirmed that **1_Zn_** is a very useful tool for the conjugation of biological molecules to transition metals. Using this compound, it was possible to obtain the zinc folate complexes **2_Zn_** and **3_Zn_** with a simple and convenient reaction, in which the products can be isolated as pure products of the reaction medium by precipitation.

In addition, the study demonstrates the viability of the transmetallation reaction between bioconjugated zinc complexes and the technetium tricarbonyl cation to prepare Tc-99m compounds, which could be used for radiopharmaceutical purposes. Tc-99m folates **2_Tc_** and **3_Tc_** were prepared with high yields from **2_Zn_** and **3_Zn_** in a reaction medium that contains an extremely low concentration of the zinc reagents (lower than 1 ppm). The structures of **2_Tc_** and **3_Tc_** were corroborated by comparing them with the HPLC data of their homologous rhenium complexes **2_Re_** and **3_Re_**. Therefore, it was concluded that this approach based on the transmetallation reaction with low soluble zinc complexes makes it possible to prepare Tc-radiopharmaceuticals with high molar activity in a simple one-pot reaction at a low temperature, without a posterior purification step.

However, the in vitro and in vivo studies of Tc-99m folates **2_Tc_** and **3_Tc_** have shown a low specific uptake in tumor cells. This problem could be caused by the 3D molecular structure of the folate complexes that prevents its binding to the folate receptor. Further studies should be carried out to study how to modify the molecular structure, in order to increase the linker length, to improve the folate receptor recognition.

## Figures and Tables

**Table 1:** Biodistribution results of complexes **2_Tc_** and **3_Tc_** in athymic mice bearing a SKOV3 tumor at 4 h post-injection.

Organs and Tissues	2_Tc_	3_Tc_
Tumor	0.58 ± 0.06	0.48 ± 0.11
Intestine	52.16 ± 4.21	38.85 ± 1.41
Pancreas	0.13 ± 0.03	0.12 ± 0.02
Spleen	0.17 ± 0.01	0.15 ± 0.03
Kidney	10.51 ± 1.76	11.77 ± 1.87
Stomach	1.63 ± 0.10	1.49 ± 0.20
Liver	2.88 ± 0.37	2.41 ± 0.25
Heart	0.31 ± 0.05	0.24 ± 0.03
Lung	0.62 ± 0.13	0.76 ± 0.16
Blood	0.39 ± 0.07	0.67 ± 0.15
Muscle	0.14 ± 0.04	0.24 ± 0.04

Data depict the amount of tracer uptake (%ID/g) and are presented as an average from 3 animals ± SEM.

## Data Availability

Data is contained within the article and supplementary material.

## Supplementary Materials

The following are available online. Figure S1: Spectroscopic data for **2_(Zn)_** (^1^H and ^13^C NMR). Figure S1: Spectroscopic data for **2_(Zn)_** (^1^H and ^13^C NMR). Figure S2: Spectroscopic data for **2_(Zn)_** (IR). Figure S3: Spectrometric data for **2_(Zn)_** (HRMS). Figure S4: Spectroscopic data for **3_(Zn)_** (^1^H and ^13^C NMR). Figure S5: Spectroscopic data for **3_(Zn)_** (IR). Figure S6: Spectrometric data for **3_(Zn)_** (HRMS). Figure S7: Spectroscopic data for **2_(Re)_** (^1^H and ^13^C NMR). Figure S8: Spectroscopic data for **2_(Re)_** (^31^P). Figure S9: Spectroscopic data for **2_(Re)_** (IR). Figure S10: Spectrometric data for **2_(Re)_** (HRMS). Figure S11: Spectroscopic data for **3_(Re)_** (^1^H and ^13^C NMR). Figure S12: Spectroscopic data for **3_(Re)_** (^31^P). Figure S13: Spectroscopic data for **3_(Re)_** (IR). Figure S14: Spectrometric data for **3_(Re)_** (HRMS).

## Sample Availability

Samples of the compounds are not available from the authors.
