# Supplementary material for: New Bioconjugated Technetium and Rhenium Folates Synthesized by Transmetallation Reaction with Zinc Derivatives
Source: Molecules. 2021 Apr 19;26(8):2373. doi: 10.3390/molecules26082373 (PMC8074163; doi:10.3390/molecules26082373)
Supplement: Supplementary file 1 [file molecules-26-02373-s001.zip › molecules-1173678-supplementary.pdf]

Supplementary Material

New Bioconjugated Technetium and Rhenium Folates synthesized by Transmetallation Reaction with Zinc Derivatives

Jordi Borràs [1], Julie Foster [2], Roxana Kashani [2], Laura Melendez-Alafort [3], Jane Sosabowski [2], Joan Suades [1,*] and Ramon Barnadas-Rodríguez [4,*]

1 Departament de Química, Edifici C, Universitat Autònoma de Barcelona, 08193 Cerdanyola del Vallès, Catalonia, Spain; jborrasamoraga@gmail.com (J.B.)

2 Barts Cancer Institute, Queen Mary University of London, John Vane Science Centre, Charterhouse Square, London, United Kingdom; j.m.foster@qmul.ac.uk (J.F.); roxana.kashani@qmul.ac.uk (R.K.); j.k.sosabowski@qmul.ac.uk (Ja.S.)

3 Istituto Oncologico Veneto IRCCS, 35128 Padova, Italy; laura.melendezalafort@iov.veneto.it

4 Unitat de Biofísica/Centre d'Estudis en Biofísica, Facultat de Medicina, Departament de Bioquímica i Biologia Molecular, Universitat Autònoma de Barcelona. 08193 Cerdanyola del Vallès, Catalonia, Spain.

* Correspondence: joan.suades@uab.cat (Jo.S.); ramon.barnadas@uab.cat (R.B.)

CONTENTS

Figure S1. Spectroscopic data for $\mathbf{2}_{(Zn)}$ (^{1}H and ^{13}C NMR) S2
Figure S2. Spectroscopic data for $\mathbf{2}_{(Zn)}$ (IR) S3
Figure S3. Spectrometric data for $\mathbf{2}_{(Zn)}$ (HRMS) S4
Figure S4. Spectroscopic data for $\mathbf{3}_{(Zn)}$ (^{1}H and ^{13}C NMR) S5
Figure S5. Spectroscopic data for $\mathbf{3}_{(Zn)}$ (IR) S6
Figure S6. Spectrometric data for $\mathbf{3}_{(Zn)}$ (HRMS) S7
Figure S7. Spectroscopic data for $\mathbf{2}_{(Re)}$ (^{1}H and ^{13}C NMR) S8
Figure S8. Spectroscopic data for $\mathbf{2}_{(Re)}$ (^{31}P) S9
Figure S9. Spectroscopic data for $\mathbf{2}_{(Re)}$ (IR) S9
Figure S10. Spectrometric data for $\mathbf{2}_{(Re)}$ (HRMS) S10
Figure S11. Spectroscopic data for $\mathbf{3}_{(Re)}$ (^{1}H and ^{13}C NMR) S11
Figure S12. Spectroscopic data for $\mathbf{3}_{(Re)}$ (^{31}P) S12
Figure S13. Spectroscopic data for $\mathbf{3}_{(Re)}$ (IR) S12
Figure S14. Spectrometric data for $\mathbf{3}_{(Re)}$ (HRMS) S13

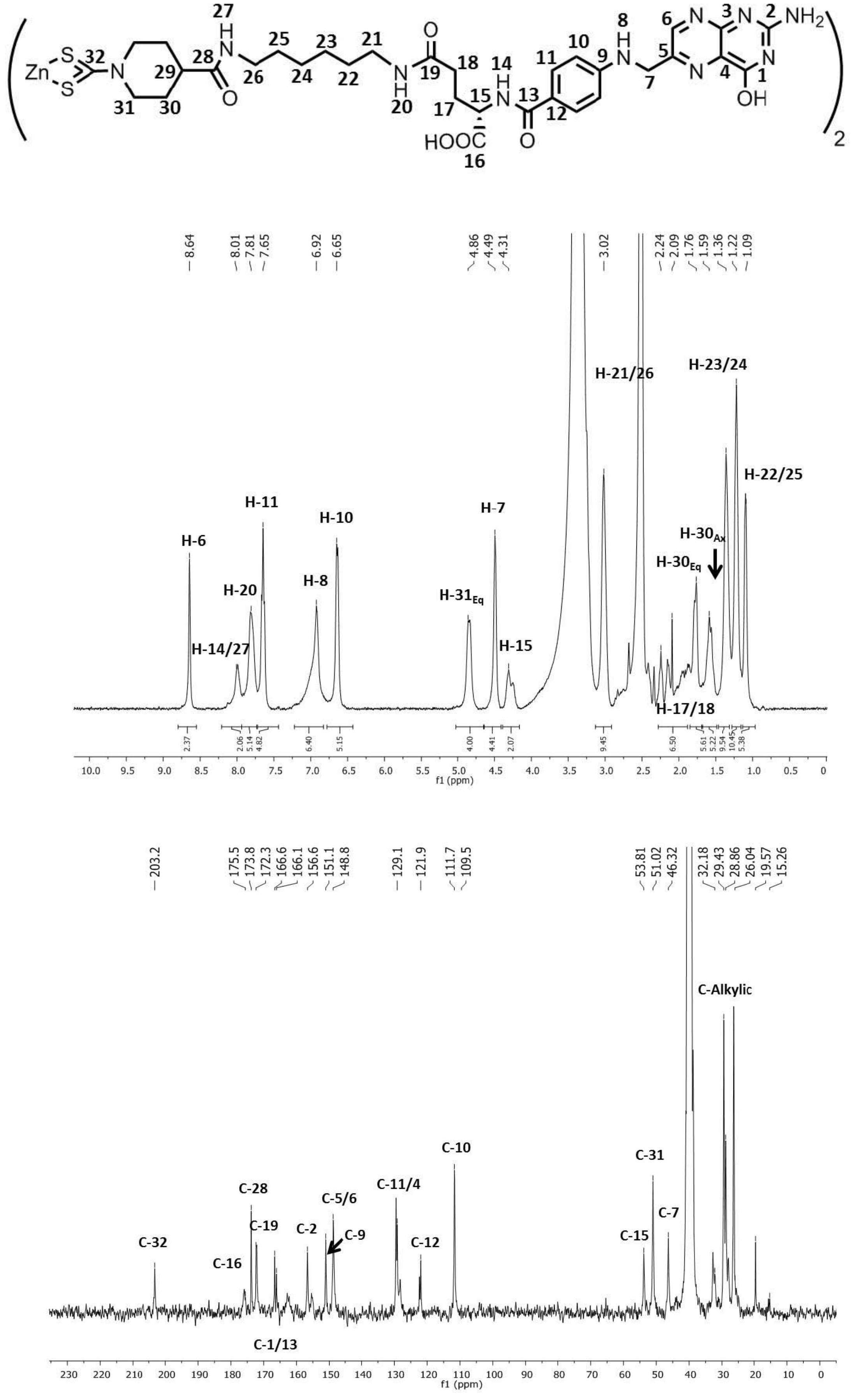

Figure S1. Spectroscopic data for **2**(Zn). ^{1}H NMR (top; d6-DMSO-400 MHz) and ^{13}C NMR (bottom; d6-DMSO-400 MHz).

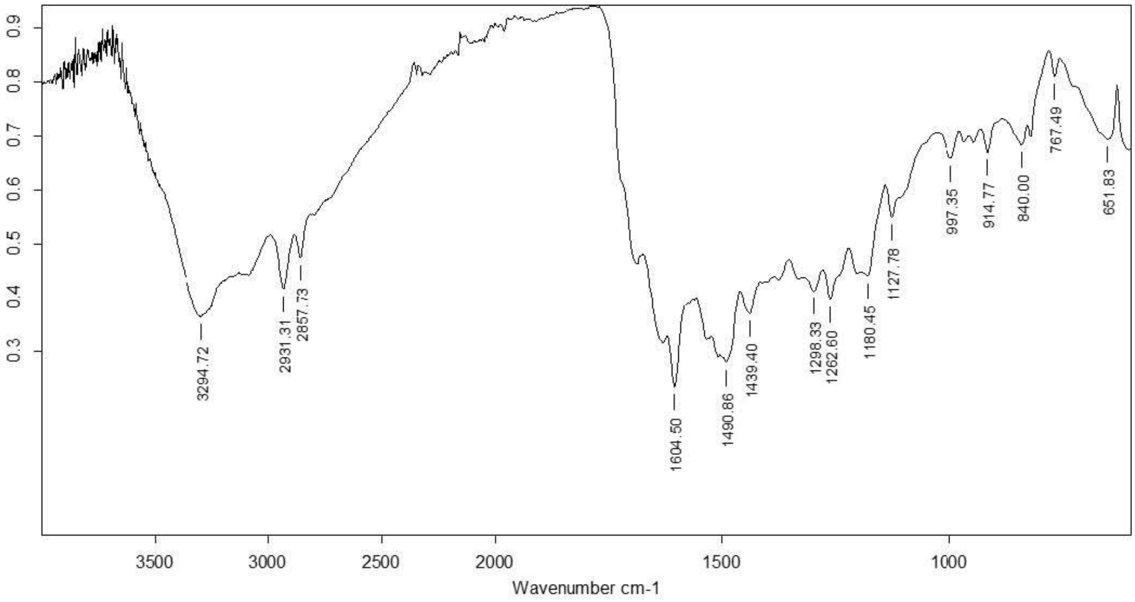

Figure S2. Spectroscopic data for **2**(Zn) (IR-ATR)

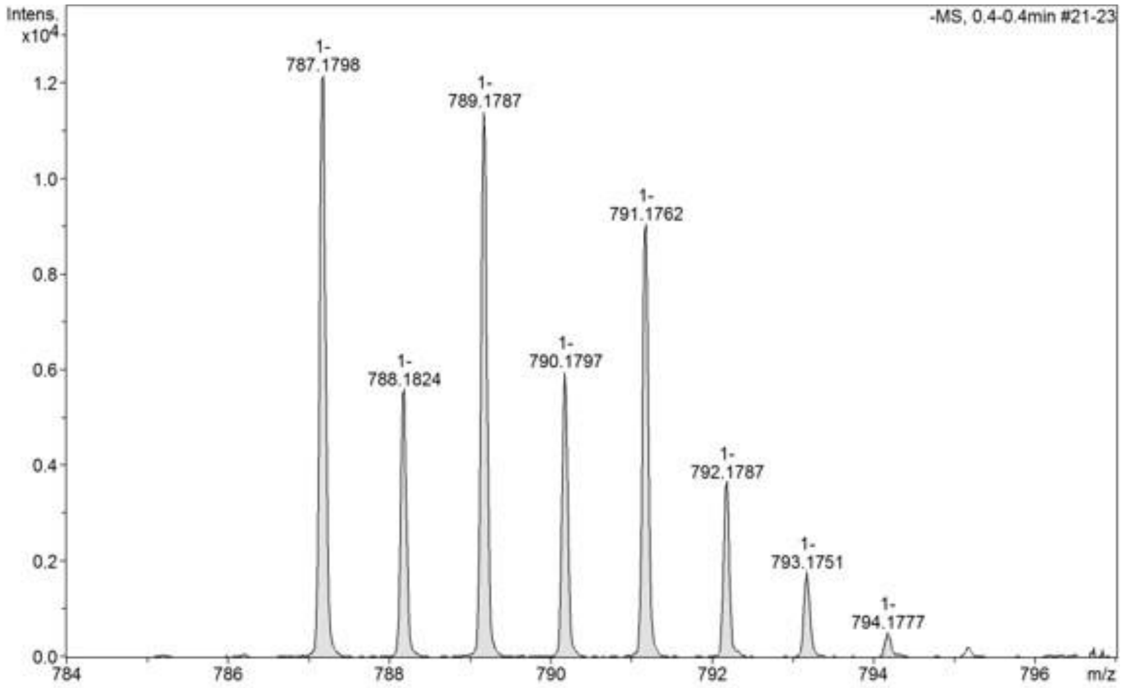

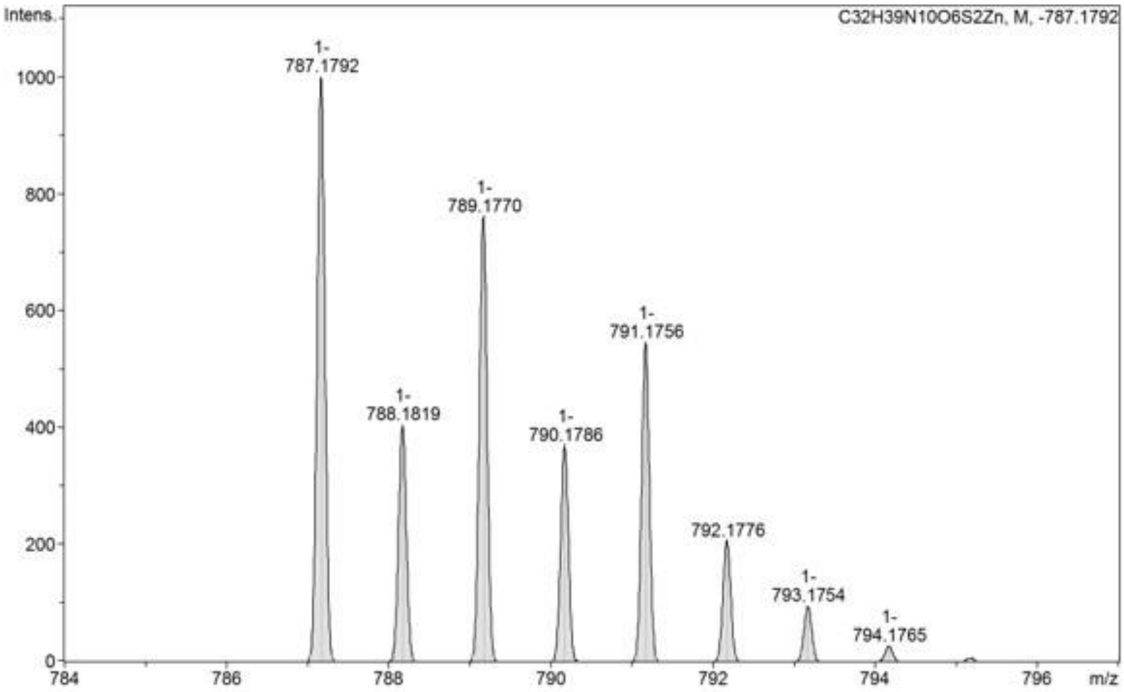

Figure S3. Spectrometric data for **2**(Zn) (HRMS). Experimental (top) and calculated (bottom) for [M]-L^{-}-$2H^{+}$.

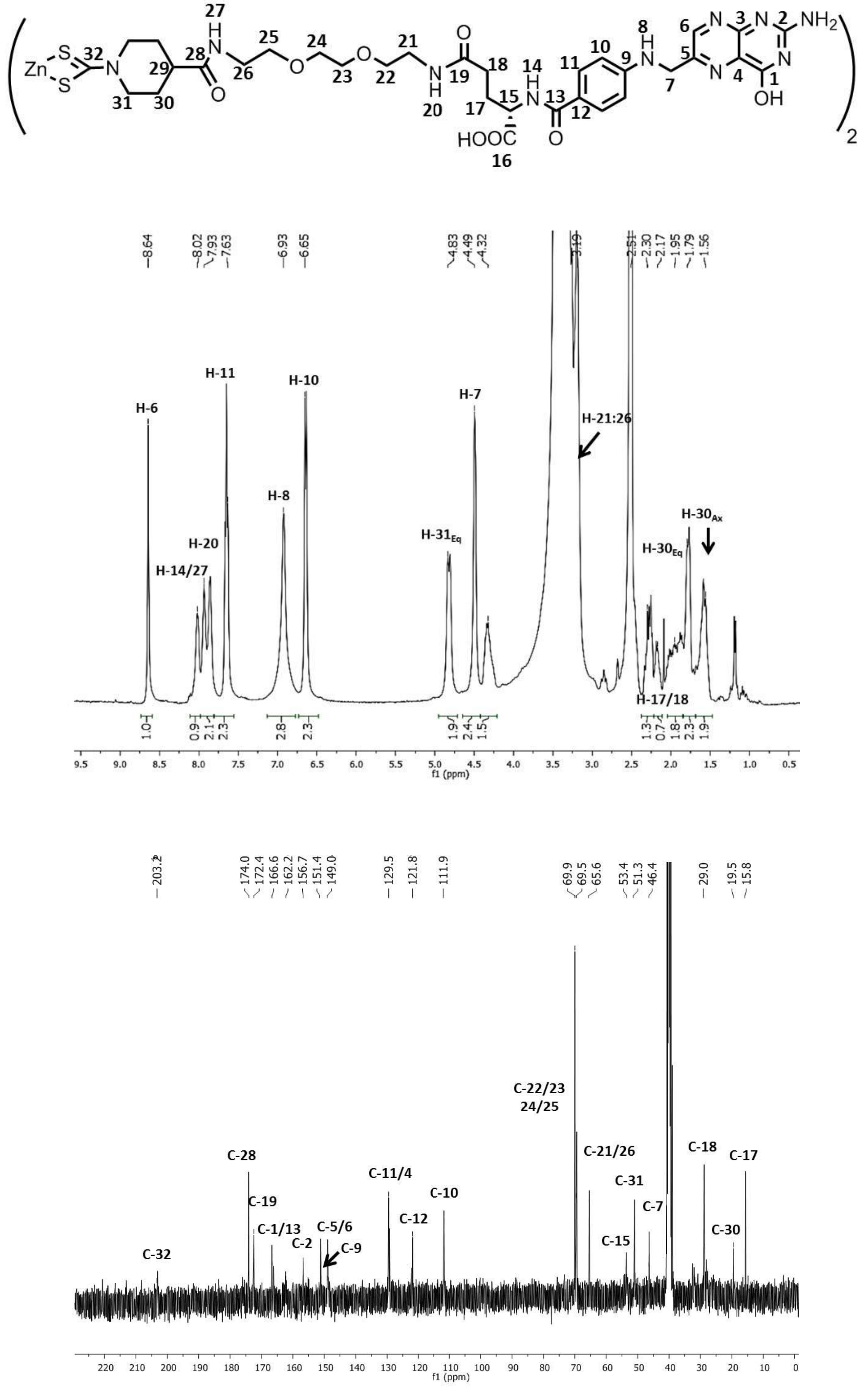

Figure S4. Spectroscopic data for **3**(Zn). ^{1}H NMR (top; d$_6$-DMSO-400 MHz) and ^{13}C NMR (bottom; d$_6$-DMSO-400 MHz).

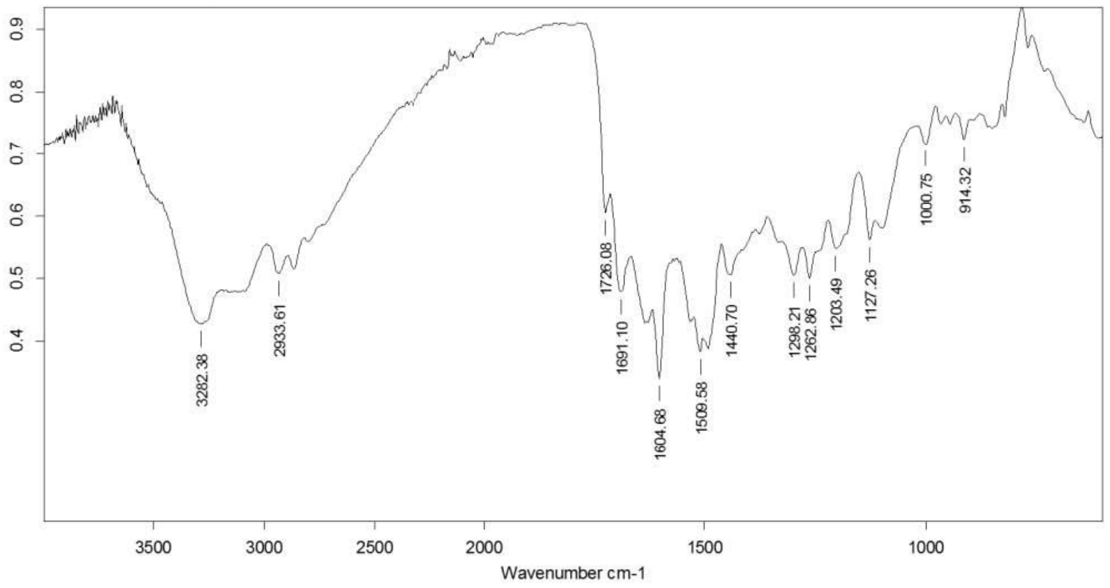

Figure S5. Spectroscopic data for **3**$_{(Zn)}$ (IR-ATR)

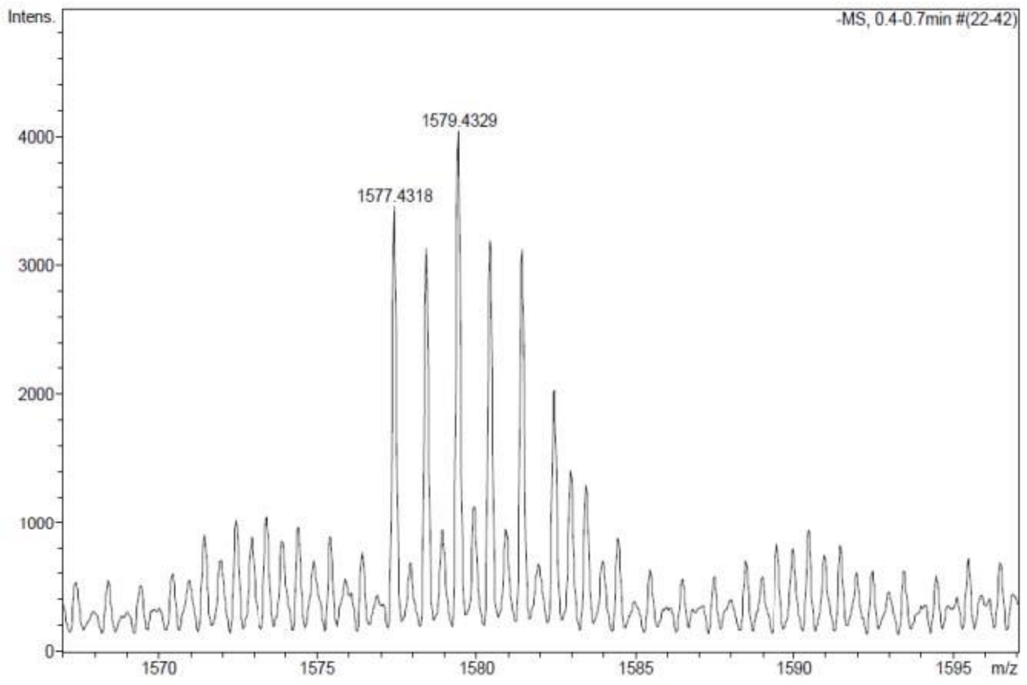

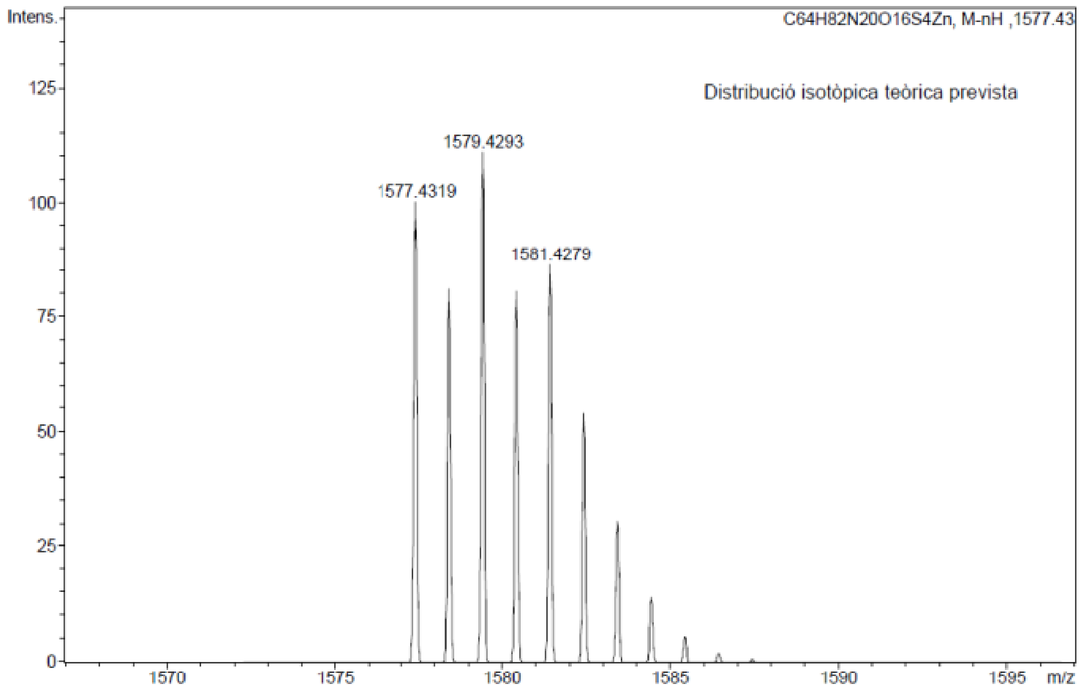

Figure S6. Spectrometric data for **3$_{(Zn)}$** (HRMS). Experimental (top) and calculated (bottom) for [M]-H^+.

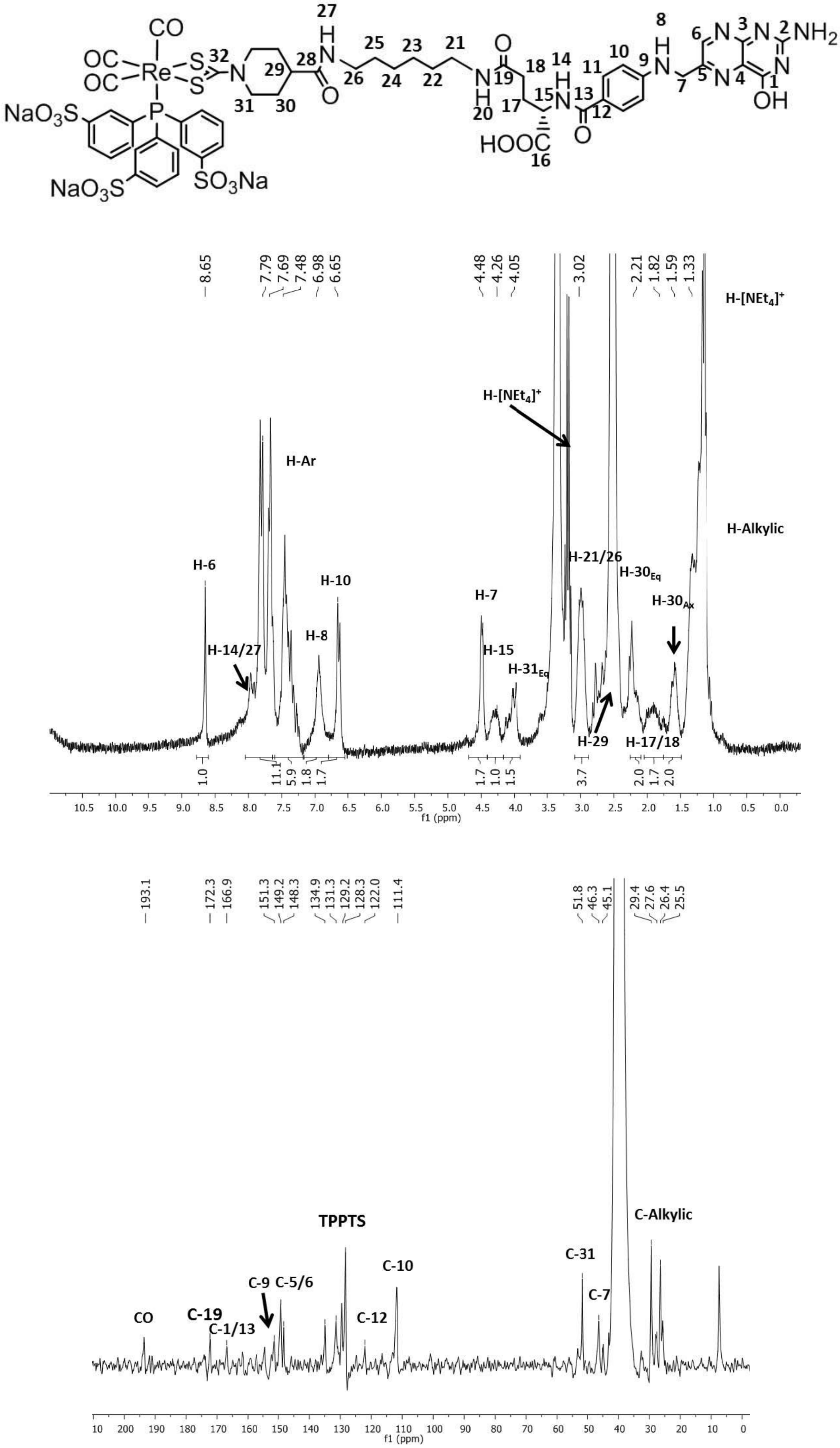

Figure S7. Spectroscopic data for **2(Re)**. ^{1}H NMR (top; d_6-DMSO-250 MHz) and ^{13}C NMR (bottom; d_6-DMSO-250 MHz).

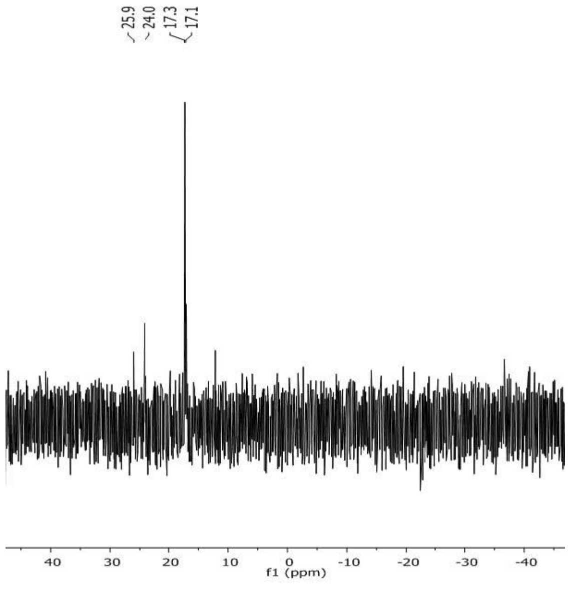

Figure S8. Spectroscopic data for **2(Re).** ^{31}P (d$_6$-DMSO-250 MHz).

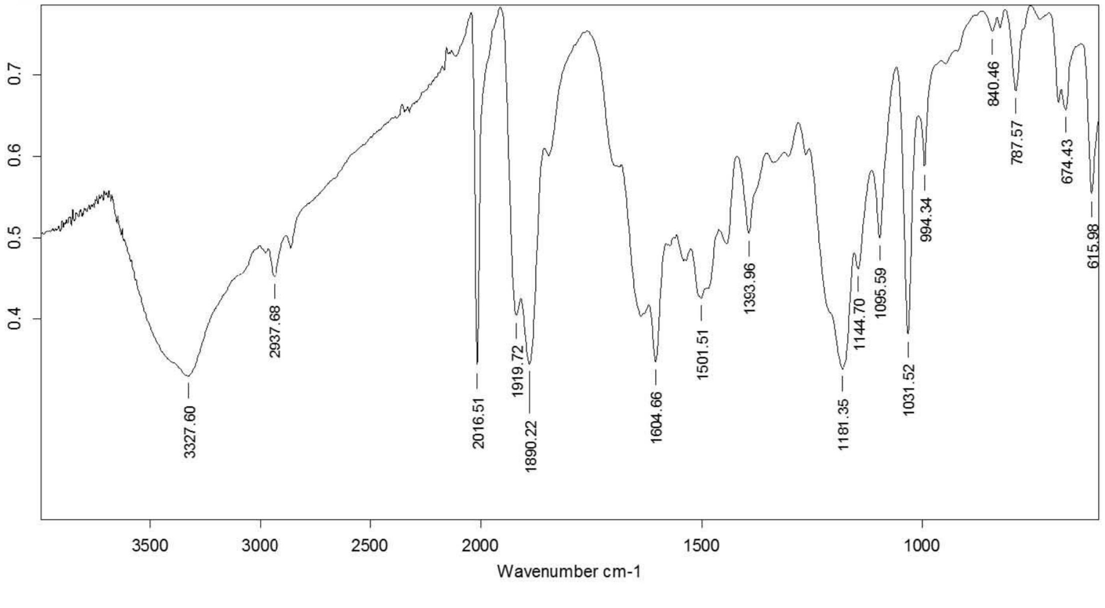

Figure S9. Spectroscopic data for **2(Re)** (IR-ATR)

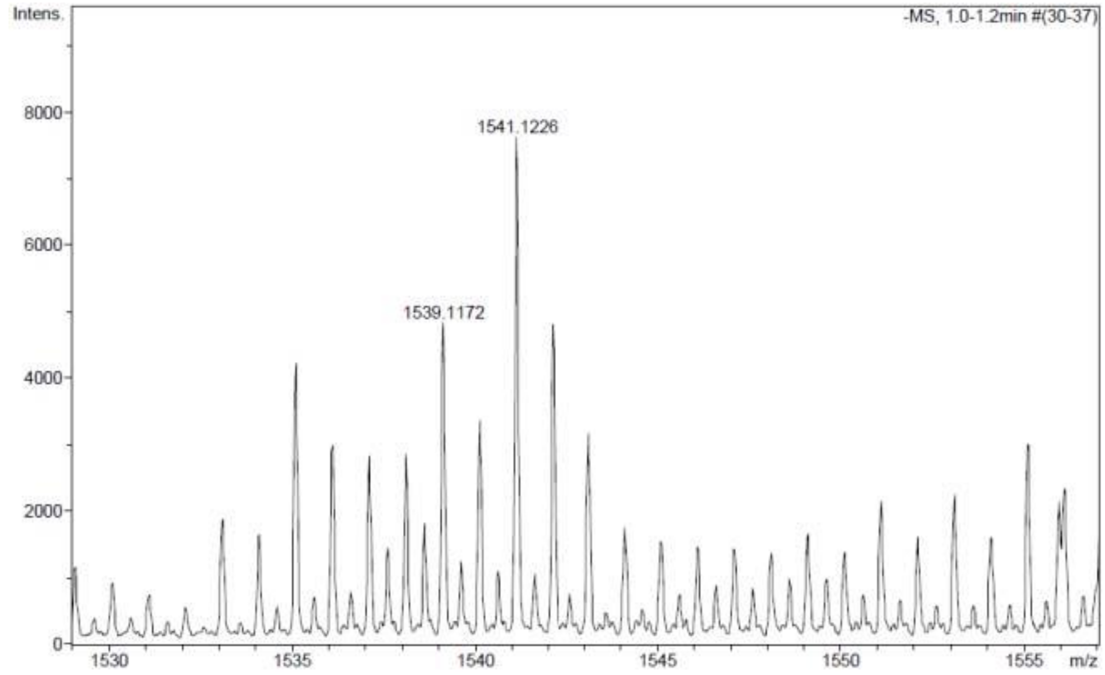

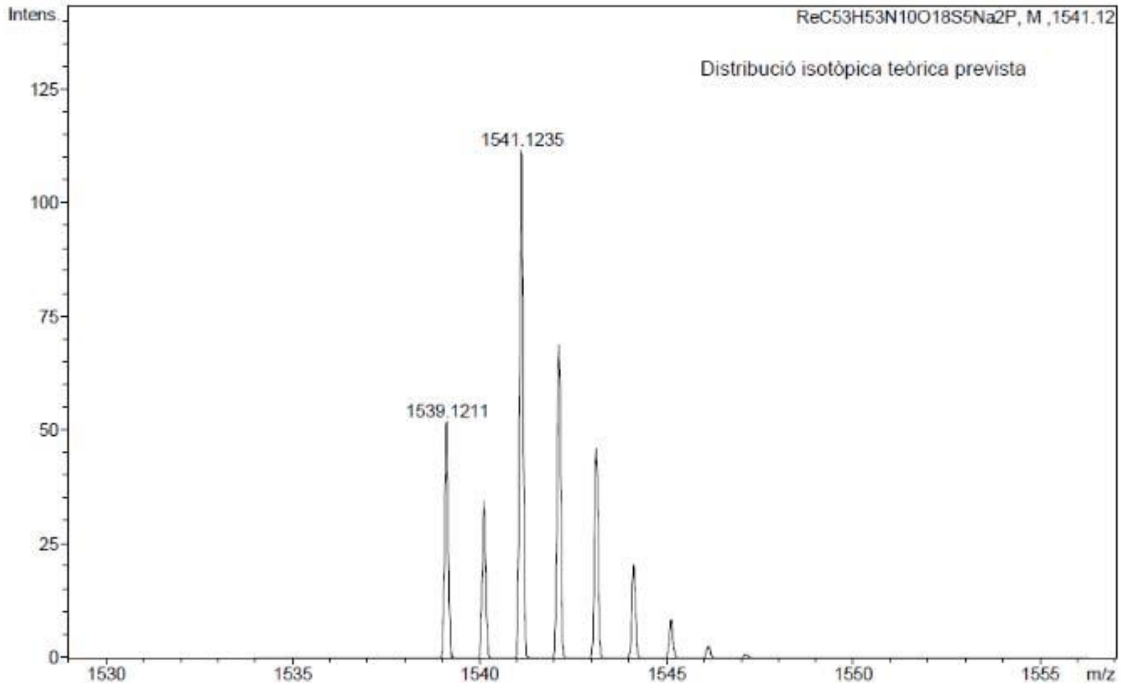

Figure S10. Spectrometric data for **2(Re)** (HRMS). Experimental (top) and calculated (bottom) for [M]-Na^+.

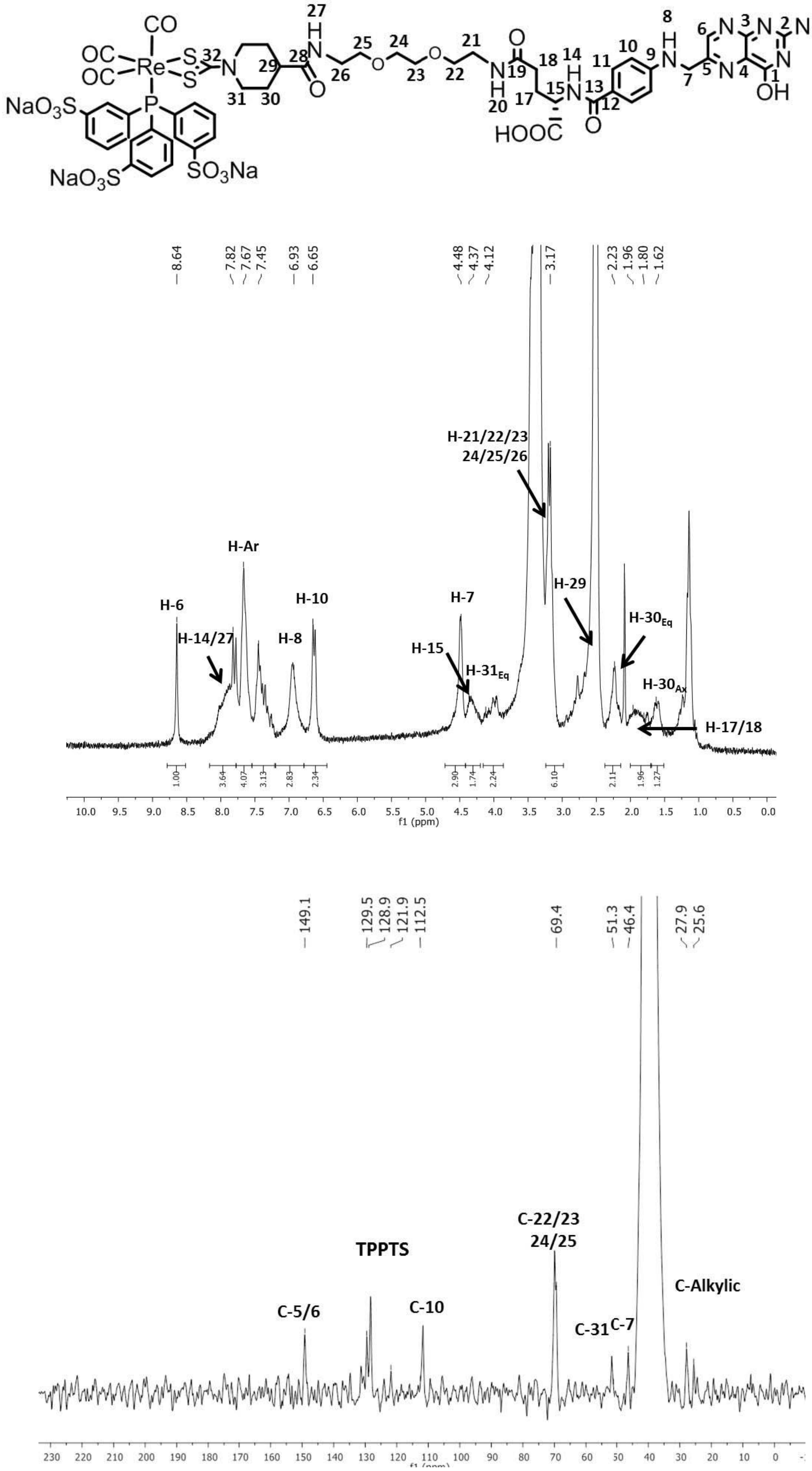

Figure S11. Spectroscopic data for **3**(Re). ^{1}H NMR (top; d6-DMSO-250 MHz) and ^{13}C NMR (bottom; d6-DMSO-360 MHz).

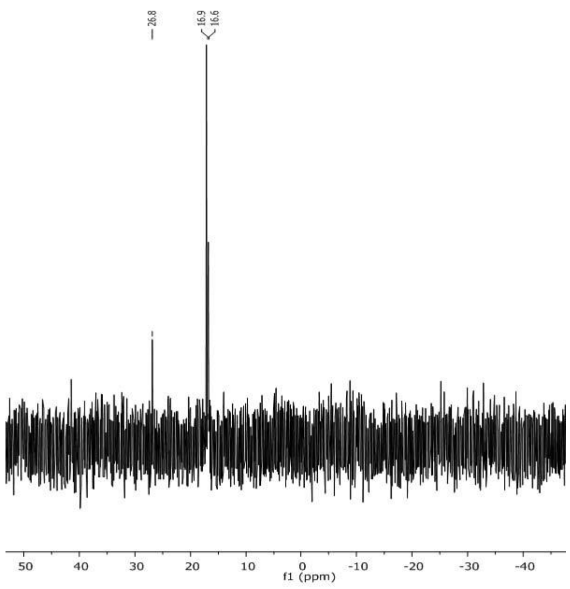

Figure S12. Spectroscopic data for **3**(Re). ^{31}P (d_6-DMSO-250 MHz).

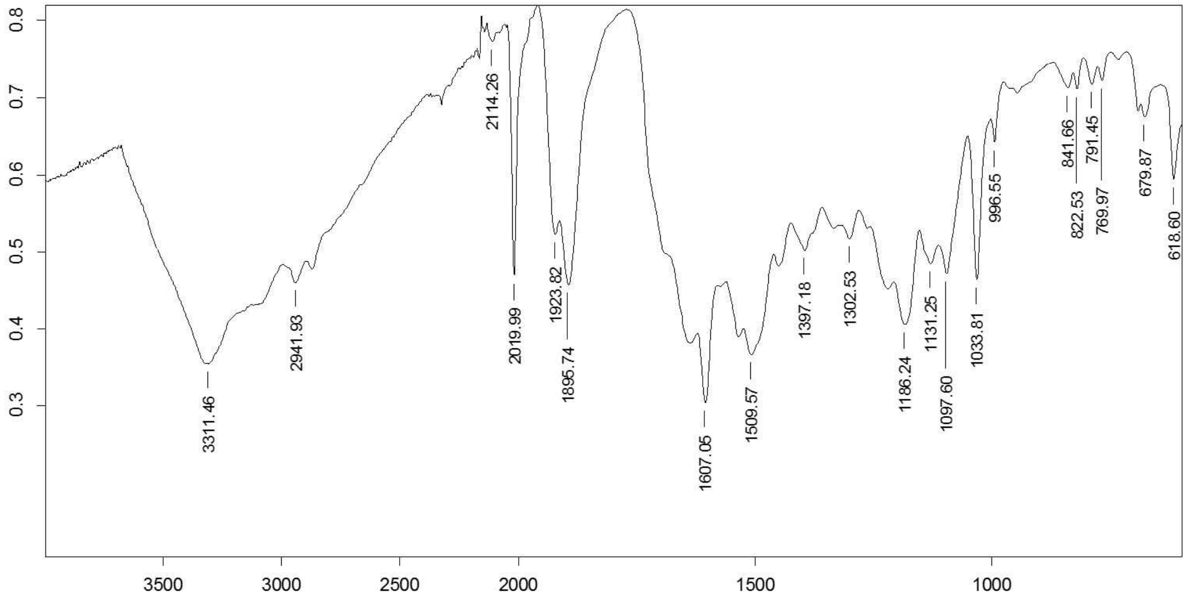

Figure S13. Spectroscopic data for **3**(Re) (IR-ATR).

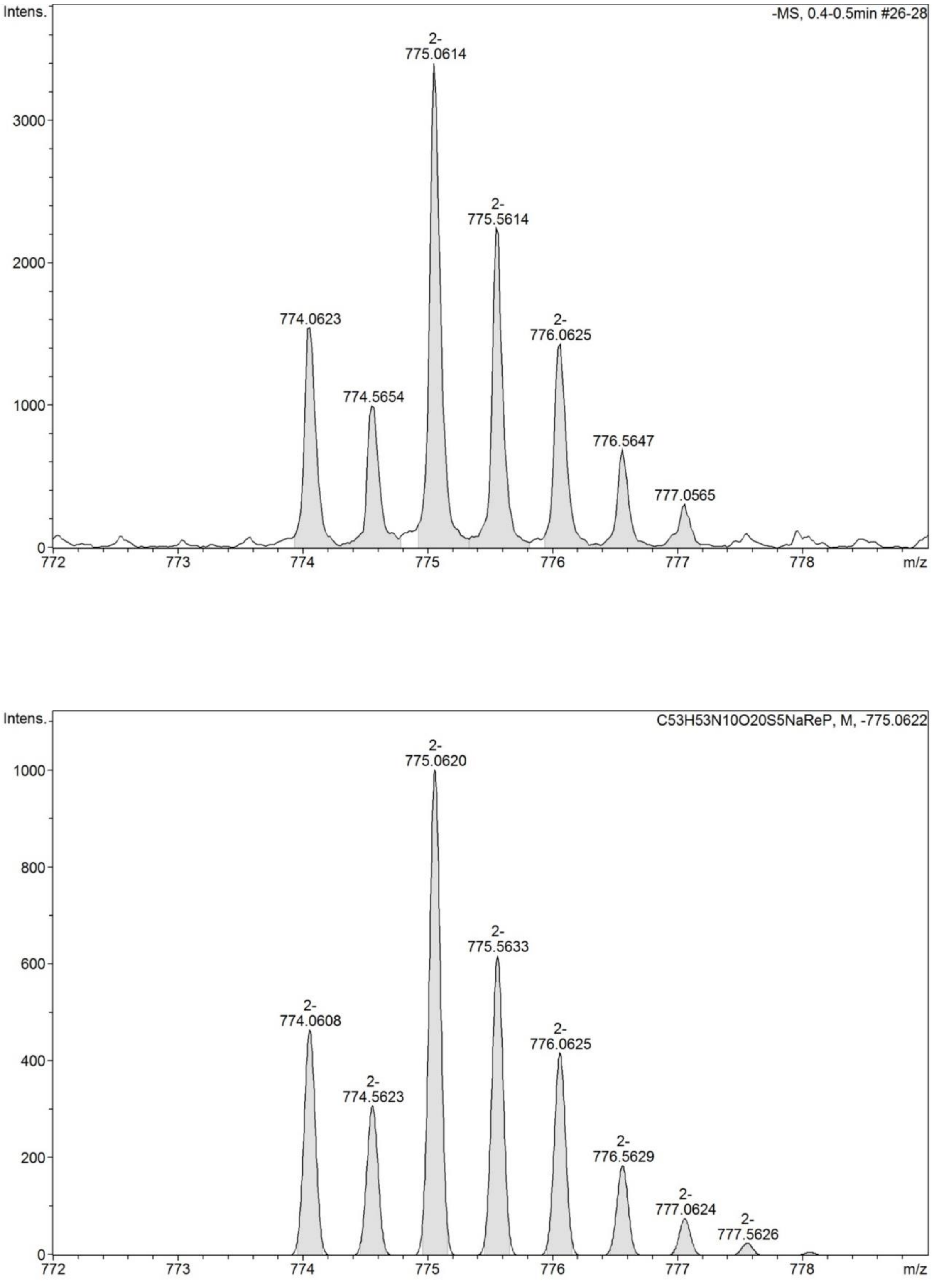

Figure S14. Spectrometric data for **3(Re)**. Experimental (top) and calculated (bottom) for [M]-2Na$^+$.
